# Review of recent advances in chitosan- and hyaluronic-acid-based wound dressings: fabrication, application, and types

**DOI:** 10.3389/fphar.2026.1795940

**Published:** 2026-06-03

**Authors:** Ling Li, Chunying Ma, Xiangyu Wu, Yunxin Han, Tanwenchen Wang, Zhensheng Li

**Affiliations:** 1 College of Science, Hainan Tropical Ocean University, Sanya, China; 2 Department of New Drug Research and Development, Institute of Materia Medical, Chinese Academy of Medical Sciences & Peking Union Medical College, Beijing, China

**Keywords:** chitosan, film, hyaluronic acid, hydrogel, nanofiber, scaffold, sponge, wound healing

## Abstract

Considering the enormous scale of the global wound dressing market, optimized wound dressings have tremendous market prospects. Chitosan (CS) shows excellent antibacterial and hemostatic activity, and hyaluronic acid (HA) has good biocompatibility, hygroscopicity, and anti-inflammatory activity. For these reasons, both are widely used as wound dressings. In this review, we systematically discuss the characteristics, bioactivities, and commercial wound healing products involving CS and HA. Their limitations, including the low water solubility of CS, instability of HA, and molecular weight (MW)-dependent anti-inflammatory activity of HA, are also outlined. Several chemically modified CS derivatives with enhanced water solubility are discussed. We summarized modified HAs and their stability, finding that mixtures of HAs with different MWs reconcile the opposing effects of low- and high-MW HAs in inflammation and angiogenesis. Moreover, we highlight several types of CS- and HA-based wound dressings, including hydrogels, nanofibers, films, sponges, and scaffolds. Their fabrication and crosslinking methods are introduced in detail, and several limitations of preparation methods and clinical applications are discussed. Finally, we discuss the challenges associated with the clinical translation of wound dressings and possible improvements.

## 1 Introduction

The skin is the body’s largest organ, situated between the body and the external environment (Ji et al., 2022a). It can protect the body against harmful external factors (Liang et al., 2016), excrete and secrete (Nestle et al., 2009), transfer gas (Ehrhardt, 2018), regulate body temperature (Nyein et al., 2021), and sense external stimuli (Chortos et al., 2016). Skin injuries lead to significant breaches that disrupt the functions of the skin (Sorg et al., 2017). Skin wounds can affect subcutaneous tissue, nerves, tendons, muscles, vessels, and bones (Okur et al., 2020), and wound infections can lead to morbidity and impose a high-cost burden (Ashraf et al., 2009). Therefore, adequate wound care is crucial for restoring the normal physiological functions of the skin. Based on the statistics and forecasts reported by QY Research, the annual turnover of the wound care market was USD 29 billion in 2023; notably, the market annual turnover of is projected to reach USD 36.8 billion by 2030, with a compound annual growth rate of 3.4% from 2024 to 2030. Within the wound care market, the global wound dressing market reached USD 14.2 billion in 2023 and is expected to reach USD 19 billion in 2030, with a compound annual growth rate of 4.14% from 2024 to 2030. These data indicate that wound dressings constitute a crucial component of wound care. Data Bridge Market Research has estimated that the annual turnover in the Asia-Pacific region will experience substantial growth due to aging populations, increased healthcare spending, expanding healthcare infrastructure, and a large patient pool. As the aging population and incidence of chronic diseases such as diabetes increase, the market for advanced wound dressings incorporating bioactive materials and sophisticated technologies will expand. Therefore, advanced wound dressings that can promote faster healing and reduce the risk of infections have garnered increased attention from research institutes and companies (Loo et al., 2022).

**FIGURE 1:**
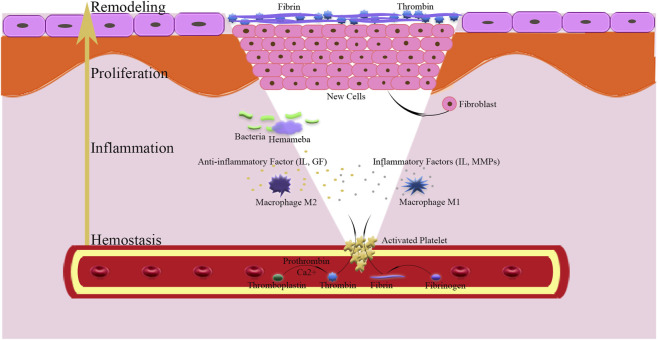
Wound healing process. In the hemostasis stage, excessive blood loss is prevented by the almost immediate formation of a platelet plug (Rodrigues et al., 2018); thrombin is synthesized from thromboplastin, Ca^2+^, and prothrombin, and fibrinogen is converted into fibrin. In the inflammation stage, M1 macrophages release pro-inflammatory cytokines, including interleukin (IL)-6 and matrix metalloproteinases (MMPs) (Krzyszczyk et al., 2018), while M2 macrophages release anti-inflammatory cytokines, including IL-10 and growth factor (GF) (Krzyszczyk et al., 2018); hemameba inhibits invading bacteria and dead cells. In the proliferation stage, collagen synthesized by fibroblasts facilitates the generation of new cells and recruits them to cover the wound area (desJardins-Park et al., 2018), while thrombin and fibrin gather on the wound surface. In the remodeling stage, new skin tissues and blood vessels gradually mature (Han et al., 2017).

Wound healing begins immediately after a skin injury (Prasathkumar and Sadhasivam, 2021). The process of wound healing has been shown to follow four sequential stages (Figure 1), namely, hemostasis, inflammation, proliferation, and remodeling (Prasanna et al., 2018). During the stages of wound healing, optimal wound care is critical for achieving hemostasis, accelerating inflammatory responses, and promoting cell proliferation and tissue remodeling (Ji et al., 2022b). Optimal wound care is usually achieved using an active wound dressing with properties that promote wound healing. Modern wound dressings use several naturally bioactive materials (Sun et al., 2020) with antimicrobial, biocompatible, anti-inflammatory, and antioxidant properties, producing materials that mimic the extracellular matrix (ECM), promote cell migration and proliferation, increase water retention, promote tissue regeneration, and absorb exudates (Sun et al., 2020; Wang K. et al., 2020; Fan et al., 2020; Bakhsheshi-Rad et al., 2019; Chen et al., 2018; Miguel et al., 2019; Kanikireddy et al., 2020). Depending on the specific properties of natural biomaterials, several types of wound dressings have been developed, including hydrogels (Asadi et al., 2021), nanofibers, films (Balasubramaniam et al., 2020), sponges (Klinger et al., 2019), and scaffold-based dressings. Of these bioactive natural materials, chitosan (CS) has been found to be an ideal choice for dressings due to its remarkable antibacterial effects (Jiang et al., 2015), antioxidant activity (Sun B. et al., 2019), biocompatibility (Hao et al., 2020), and biodegradability (Ji et al., 2022a). Notably, CS shows better antibacterial effects than other biopolymers. The amine groups in CS can be protonated in acidic solution, and CS derivatives such as quaternized CS show good water solubility. The protonated amines of CS derivatives can interact with anionic bacteria and disrupt their functions. CS and its derivatives have, thus, become the standard biopolymers for preparing materials with inherent antibacterial properties (Liu et al., 2022). In contrast, other anionic or neutral biopolymers usually need additional antibacterial agents. In addition, CS-based biomaterials exhibit good hemostatic effects (Mecwan et al., 2022).

Hyaluronic acid (HA), primarily distributed in connective tissue, is an important component of the ECM and can trap water (Castro et al., 2021), adhere to cells, promote proliferation and migration, and form granulation tissue (Saha and Rahi, 2021; Sionkowska et al., 2020). Notably, HA is in the connective, epithelial, and neural tissues of the human body and shows good biocompatibility and hygroscopicity (Voigt and Driver, 2012). Although collagen and gelatin also reveal good biocompatibility, they show weaker hygroscopicity than HA. In addition, HA plays very important roles in the wound healing process. HA provides a temporary structure that facilitates the diffusion of nutrients and the removal of waste products. HA can also promote the proliferation and migration of keratinocytes, which are crucial for wound healing (Voigt and Driver, 2012). Therefore, this review summarizes CS and HA in different types of wound dressing: hydrogels, nanofibers, films, sponges, and scaffold-based dressings.

## 2 Wound dressings

**FIGURE 2:**
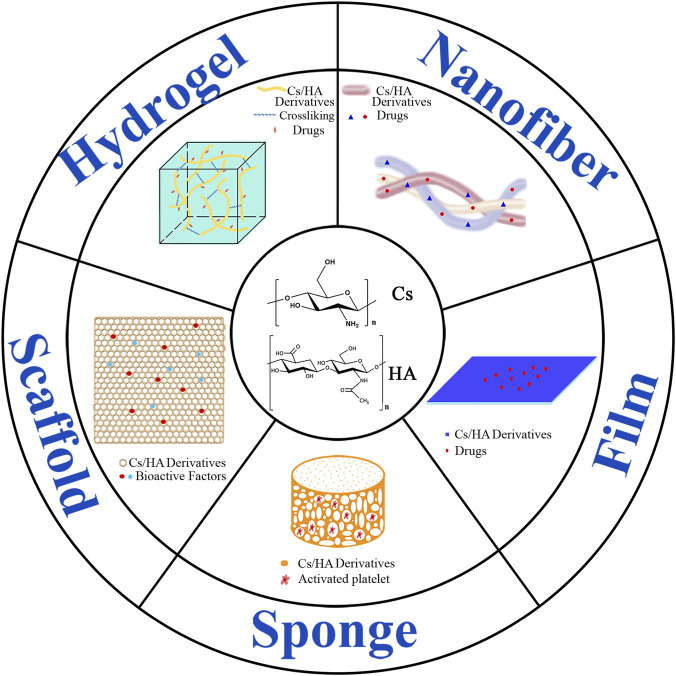
Different types of wound dressings based on CS and HA. CS- and HA-derivative hydrogels are formed through physical or chemical crosslinking and loaded with drugs. CS- and HA-derivative nanofibers and films are loaded with drugs. CS- and HA-derivative sponges are loaded with activated platelets. CS- and HA-derivative scaffolds are loaded with bioactive factors.

Modern wound therapies include negative pressure therapy (Everett and Mathioudakis, 2018), hyperbaric oxygen therapy (Thom, 2011), skin grafts (Serra et al., 2017), and ultrasound therapy (Jahromi et al., 2018). However, wound dressings are still the most commonly used therapy for wound care (Ji et al., 2022a). The primary purpose of wound dressings is to protect the wound during the first 48 h after injury, while sufficient barrier functions of the injured wound are being established (Toon et al., 2013). Therefore, the objectives of wound dressings include protecting the healing wound bed (Kelly-O'Flynn et al., 2020), promoting hemostasis around the wound (Sabab et al., 2021), minimizing wound pain, and absorbing exudates (Holloway and Bradbury, 2024). In the next stage of wound healing, granulation tissue, epithelium, and blood vessels begin to form. The characteristics of wound dressings needed during the proliferation stage include the promotion of granulation tissue formation, management of local or systemic infection, and the maintenance of a moist wound environment (Holloway and Bradbury, 2024; Abd El-Hack et al., 2020; Moeini et al., 2020; Tao et al., 2020). Optimal wound dressings promote hemostasis, collagen deposition, angiogenesis, and rapid wound closure; they exhibit antimicrobial, anti-inflammatory, and antioxidant activity (Loo et al., 2022). Based on these requirements, several types of effective wound dressings (hydrogels, nanofibers, films, sponges, and scaffolds) based on CS and HA have been the focus of research (Figure 2).

## 3 CS-based wound dressings

### 3.1 CS and its derivatives

CS, the only known natural cationic polysaccharide (Sun B. et al., 2019), demonstrates good antibacterial, hemostatic, and antioxidative activity. The antibacterial activity of CS is primarily due to its positive charges. The protonated amine of CS can interact with anionic bacteria and disrupt the function of their cytoderm, cytomembrane, and proteins (Li et al., 2023). The antibacterial activity of CS has been shown to increase alongside its water solubility and molecular weight (MW) (Chang et al., 2015). CS with a MW between 3.3 and 300 kDa was found to dissolve in acid–water solutions at pH 5.0–6.0 (Panda et al., 2019). The antibacterial activity of CS increases as its MW increases, while the antibacterial activity of CS with an MW > 29.2 kDa was found to sharply decrease due to a sharp decrease in water solubility (Kou et al., 2022). Moreover, CS with excellent hemostatic activity has been applied in clinical CS wounds as a CS hemostatic sponge. The possible mechanisms of hemostatic activity have been reported as follows. 1) CS triggered red blood cell (RBC) agglutination, forming a hemostatic plug at the bleeding site due to interaction between cationic CS with anionic RBC membranes (Jin et al., 2018). 2) CS activated platelets, inducing aggregation. 3) CS derivatives readily interacted with blood cells and plasma proteins, forming a polymer–blood 3D network that blocked further blood loss (Zhang et al., 2024). The remarkable antioxidant activity of CS has been attributed to its intra-molecular hydrogen bonding effects (Anraku et al., 2018) and its water solubility, which increases its access to free radicals, thereby increasing free radical scavenging activity (Xing et al., 2004; Xing et al., 2005). The antioxidant activity of CS is influenced by its MW. CS with an MW < 50 kDa showed higher antioxidant activity than CS with an MW > 50 kDa (Tomida et al., 2010). CS with an MW of 38 kDa showed the best antioxidant activity among several low MW CS (22, 38, 52, and 81 kDa) (Tomida et al., 2009).

**FIGURE 3:**
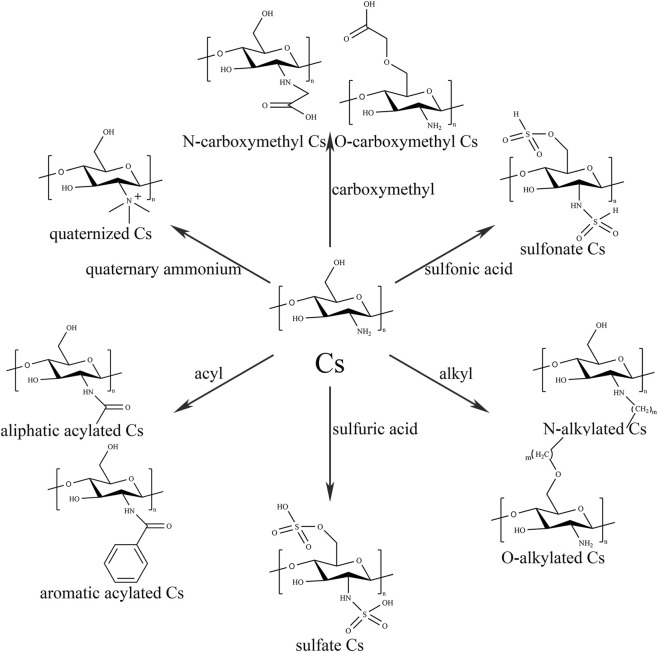
Several CS derivatives with high water solubility (carboxymethyl CS, quaternized CS, alkylated CS, acylated CS, and esterified CS).

Although CS has demonstrated good bioactivity, its water insolubility limits its clinical applications (Sang et al., 2015). CS derivatives with high water solubility have shown greater antibacterial and antioxidant properties. To improve its water solubility, CS has been modified with carboxymethyl (Hao et al., 2020), quaternary ammonium (Sun K. et al., 2019), alkyl (Sahariah and Másson, 2017), acyl (Wang K. et al., 2020), and sulfuric acid or sulfonic acid (Ramasamy et al., 2017), or it has been modified with both carboxymethyl and quaternary ammonium (Yin et al., 2018) (Figure 3). Sahariah and Másson, (2017) synthesized carboxymethyl CS and found that it showed good moisture retention, which could be modified according to the position of the substituted carboxymethyl groups. The C6-carboxymethyl group exhibited better water retention activity than the N-carboxymethyl group (Chen et al., 2002). Carboxymethyl CS has also demonstrated good biosafety as both carboxymethyl CS and its degradation product have been shown to be non-toxic and nonirritating. In addition, carboxymethyl CS efficiently retained water, inhibited *Staphylococcus aureus* and *Escherichia coli*, facilitated fibroblast proliferation, and controlled drug release. Consequently, carboxymethyl CS is widely used as a biomedical material in wound dressing and drug delivery systems and as an antibacterial agent (Fonseca-Santos and Chorilli, 2017). Carboxymethyl CS biomedical materials must comply with “YY/T 0953-2020 Medical Carboxymethyl Chitosan” in China, which stipulates requirements for appearance, solubility, molecular weight distribution, heavy metal content (Pb ≤ 10 ppm), protein residue, and degree of UV absorbency. Xue et al. (2019) synthesized quaternized CS and found that the introduced positive charges enhanced its antibacterial competence. The quaternized CS showed inhibitory activity against Gram-negative *E. coli*, Gram-positive *Listeria innocua*, and the phytopathogenic fungus *Botrytis cinerea* (Cohen et al., 2022). The antibacterial activity was influenced by its degree of substitution, with a higher degree of substitution increasing antimicrobial activity. The quaternized CS showed good biodegradability, with a low degree of substitution associated with minimal toxicity, although a high degree of substitution may have destroyed cell membranes (Peng et al., 2010). In addition, the quaternized CS exhibits antibacterial and antiviral activity, facilitates angiogenesis, enables targeted drug delivery, and enhances vaccine activity. Therefore, quaternized CS may be used as an antibacterial agent, a wound dressing, a drug delivery system, and a vaccine. In China, biomedical materials containing quaternized CS must comply with class II medical device regulations, which stipulate limits for heavy metals, protein residues, and endotoxins. Sahariah and Másson (2017) found that alkylation weakened intermolecular and intramolecular hydrogen bonds and improved the water solubility of CS. However, CS modified with long alkane chains had increased hydrophobicity. In addition, alkylated CS showed better hemostatic activity than CS as the former accelerated the gelling process (Huang et al., 2017), and the hemostasis rate increased as the alkane chains lengthened (Chen K. et al., 2017). Alkylated CS showed good biocompatibility and biosafety; therefore, alkylated CS has potential for use as a hemostatic material and drug vector. Alkylated CS biomedical materials must comply with ISO 10993 biocompatibility evaluation criteria. Wang W. et al. (2020) synthesized acylated CS by introducing aliphatic or aromatic acyl groups into CS, which showed good water solubility. Although the acyl group disrupted intramolecular and intermolecular hydrogen bonds in CS, a high degree of substitution of the acyl group decreased CS solubility (Tiew and Misran, 2017). In addition, the amphiphilic character of acylated CS contributed to strong interactions between acylated CS and mucin, enhancing antibacterial activity (Piegat et al., 2020). Moreover, the acylated CS had good biocompatibility and nontoxicity. Acylated CS has been mainly used in applications such as drug vectors, hemostatic materials, and tissue-engineered scaffolds. Acylated CS biomedical materials also must comply with ISO 10993 biocompatibility criteria. Ramasamy et al. (2017) produced esterified CS with sulfonic and sulfuric acid, and these esterified CS materials showed good water solubility, anticoagulant efficacy, and immunostimulation. Although esterified CS has demonstrated good biocompatibility, it may cause bleeding due to its anticoagulant activity. It has potential for use in antiviral, antithrombotic, drug vector, and antidiabetic applications. Esterified CS must comply with the new drug and biomaterials regulations. Chen Z. et al. (2017) developed a quaternized carboxymethyl CS to optimize multiple characteristics. The carboxymethyl groups of CS attracted Cu^2+^ and killed bacteria, and thus, the quaternized carboxymethyl CS prevented algae growth (Cai et al., 2016).

### 3.2 CS-based products for wound care

At present, CS-based biomaterials are used in commercial products for wound care (Table 1) (Biranje et al., 2021; Smith et al., 2016). HemCon®, Celox®, and Chitoflex® were analyzed for their hemostasis effects in wound healing. Granville Chapman et al. (2011) concluded that these products showed varying degrees of efficacy for managing arterial, venous, or mixed bleeding. Celox® has demonstrated significant advantages in hemostasis, particularly in battlefield settings (Ji et al., 2022a). Muzzi et al. (2012) reported on two patients who underwent post-cardiotomy extracorporeal circulatory support in which life-threatening bleeding developed because of severe coagulopathy. Celox® gauze packed on the sternal edges and pericardial cavity successfully controlled the bleeding. HemCon® was shown to be a clinically effective hemostatic dressing. Malmquist et al. (2008) reported that HemCon® dental dressing used to treat the surgical sites in patients who underwent oral surgery, including those taking oral anticoagulation therapy, achieved rapid hemostasis in under 1 min. In addition, HemCon® had favorable effects on wound healing, whether or not the wound was infected with *S. aureus* (Burkatovskaya et al., 2008). However, the clinical application of HemCon® is limited because it is strongly influenced by the wound conditions. Wounds that are too dry cause the dressing to tear easily, while very wet conditions can cause excessive dressing gelation (Muzzarelli et al., 2007). The 3M product Tegasorb® absorbs exudates, and Chitopack C® improves wound healing and rebuilds normal subcutaneous tissue (Valachova et al., 2020).

**TABLE 1:** Manufacturer, form, and characteristics of commercial CS-based wound dressings.

Product	Manufacturer	Form	Characteristics
Chitosan skin®	Hainan Xinlong Nonwovens Co., Ltd., China	​	Skin substitute
Chitosan electrospun layers™	Advanced BioMatrix, United States	Nanofibrous film	Cell culture and tissue engineering
Chitopack C®	Eisai Co., Ltd., Tokyo, Japan	Sponge	Subcutaneous tissue reconstruction, skin regeneration
Chitodine®	IMS, Malabo, Guinea	Powder	Skin wound disinfection and cleaning through adsorbed iodine
Chitoflex®	Tricol Biomedical, United States	Sponge	Strong wound surface adhesion, easy removal
Chitoseal®	Abbott Vascular, United States	Topical hemostasis pad	Hemostasis at vascular access sites and percutaneous catheters
Chitopoly®	Fuji Spinning Co., Ltd., Japan	Dressing	Antibacterial and prevents chronic dermatitis wounds
Crabyon®	Ohmikenshi	​	Comfortable ports dressing
Vulnosorb®	Tesla-Pharma	Sponge	​
Tegasorb®	3M, United States	Gel	Used in chronic and sacral wounds
BST-CarGel®	Piramal Life Sciences, Quebec, Canada	Scaffold	Hemostasis in cartilage lesion of knee and hip joints
HemCon®	HemCon Medical, United States	Dressing	Hemostasis
Traumastat®	Ore-Medix, Lebanon, United States	Porous nonwoven sheet	Surface area 100-fold more than the chitosan gauze
SyvekPatch®	Marine Polymer Tech, Inc., United States	Fibrous crystalline patch	Increased aggregation of RBCs and fibrin clot formation
ExcelArrest® XT	(Hemostasis LLC), United States	Foam hemostat pad	Accelerated hemostasis
Kyto-Cel®	Aspen Medical Europe Ltd., United Kingdom	Dressing	Removal of debridement
Celox	Medtrade Products, United States	Bandage and granular	Used in groin transection and groin puncture
HemCon® Dental dressing	Zimmer Holdings; HemCon Medical, United States	Sponge	Used in post-extraction sockets in patients under anti-platelet treatment
Clo-Sur P.A.D. and Clo-SurPLUS P.A.D	Merit Medical, United States	Nonwoven topical pad	Used to seal the radial artery access site and accelerated hemostasis

## 4 HA-based wound dressings

### 4.1 HA and its derivatives

HA is a component of the ECM (Choi et al., 2017). It provides a temporary framework for the transport of nutrients and exudates. The proliferation and migration of keratinocytes are also affected by HA (Altman et al., 2019). HAs with different MWs, from 5 × 10^5^ to 4–5 × 10^6^ Da, have demonstrated disparate effects in the inflammation stage of wound healing. High-MW HA has been shown to cause an exaggerated inflammatory response and impede wound healing, while low-MW HA promoted anti-inflammatory responses (Cui et al., 2019). The MW of HA also impacted angiogenesis in advanced wound healing applications (Fouda et al., 2016). HA promoted angiogenesis by enhancing the growth and migration of endothelial cells, while high-MW HA induced anti-angiogenic effects by slowing the migration of leukocytes and reducing excessive responses and edema (Huang and Huang, 2018). In addition, HA exhibited good antioxidant properties to suppress reactive oxygen species (ROS) in wound healing (Johnson et al., 2018). Therefore, it is crucial to reconcile the opposing effects of low- and high-MW HA. D’Agostino et al. (2019) developed a hybrid cooperative complex (HCC) of high- and low-MW HAs (ratio 1:1), with results showing that HCC reduced transforming growth factor-β, interleukin-6, and interleukin-8 levels and accelerated wound healing better than linear HA. In addition, Yamamoto et al. (2013) prepared a dressing with a two-layered spongy structure: a lower layer composed of low-MW HA and an upper layer composed of high-MW HA. The results showed that epidermal growth factor (EGF) maintained in the high-MW HA of the dressing enhanced the production of vascular endothelial and hepatocyte growth factors. Moreover, O-HA with two to ten disaccharide units accelerated angiogenesis in wound healing based on the RHAMM-mediated signaling pathways (Gao et al., 2008). Wang et al. (2016) found that O-HA facilitated human umbilical vein endothelial cell (HUVEC) migration into a wound area *in vitro*, and approximately 30% of a wound treated with O-HA was covered with HUVECs, while only 15% of a wound treated without O-HA was covered with HUVECs. Wounds in diabetic rats treated with O-HA had lower CD31 expression, enhanced blood supply and capillary density, and greater re-epithelization and granulation (Graça et al., 2020). However, HA is easily degraded *in vivo*, usually through either specific (enzymatic-hyaluronidases) or non-specific (oxidative degradation by ROS) pathways (Castro et al., 2021). In mammals, there are six hyaluronidases (HYALs): HYAL1, HYAL2, HYAL3, HYAL4, HYALP1, and PH20/SPAM1 (Castro et al., 2021). In order to improve the mechanical, rheological, and swelling activity of HA and modulate its degradation rate (Fallacara et al., 2018), HA has been modified through chemical and physical crosslinking. Six available sites on HA can be chemically modified, including a carboxyl group, four hydroxyl groups, and an N-acetyl group (Benedetti et al., 1993). The carboxyl groups can be modified into esters or amides, and the hydroxyl groups can be modified into ethers, hemiacetals, or carbamates. In order to extend its *in situ* permanence and improve its stability against HYAL, Benedetti et al. (1993) synthesized an HA benzyl ester (HYAFF 11), which also decreased the water solubility of HA. In addition, Oh et al. (2010) synthesized a functional thiol HA by conjugating HA with cystamine. According to the ring opening mechanism of maleic anhydride, Vasi et al. (2014) prepared HA–butanediol diglycidyl ether in an aqueous solution through a simple synthetic procedure. The deacetylation of the *N*-acetyl group of HA is not frequently used to obtain HA derivatives because the deacetylation reaction often induces chain fragmentation (Bellini and TOPAI, 2013; Crescenzi et al., 2002). In addition to chemical modification of HA, HA crosslinking is used to prepare HA hydrogels and improve its stability against HYALs (Zerbinati et al., 2020). Modified HA has also been chemically crosslinked using the following crosslinkers: dithiothreitol, polyethylene glycol (PEG)-dithiol, peptide linkers, and sodium tetrathionate (Saravanakumar et al., 2022). Physical crosslinking has also been used to achieve relatively high stability (Balima et al., 2024). This is typically accomplished through hydrogen bond crosslinking (Zhao et al., 2024) and metal coordination crosslinking (Prakash et al., 2024). Lee et al. (2018) used tannic acid (TA) to prepare physically crosslinked HA hydrogels. HA was first chemically crosslinked with PEG diglycidyl ether in a NaOH solution at 25 °C for 24 h. Subsequently, the HA hydrogels were immersed in different concentrations of TA solution for 2 days; during this time, physical crosslinking formed strong hydrogen bonds between the hydrogel network and the abundant hydroxyl groups of TA. Metal coordination crosslinking was achieved by metal ions that formed coordination bonds with the nitrogen and oxygen atoms in HA. Prakash et al. (2024) found that many bivalent cations, including Fe(II), Cu(II), Zn(II), Mn(II), Pd(II), Co(II), Ni(II), and particularly Mg(II), effectively crosslinked HA. Deprotonation of HA increased its negative charges, promoting interaction with the metal cations. In addition, the metal coordination-crosslinked HA hydrogels showed good stability, conductivity, and antibacterial properties (Balima et al., 2024).

### 4.2 HA-based products for wound care

The earliest HA dressing for burn therapy was developed in 1968 (Fatini et al., 1968). Presently, different HA-based wound dressings are used clinically (Table 2). HYAFF® 11 is an HA-derivative polymer obtained from the esterification product of HA with benzylic alcohol that has improved stability compared with HA while maintaining its bioactivity and biosecurity (Longinotti, 2014). When the ester bond of HYAFF® 11 undergoes hydrolytic degradation *in vivo*, the material becomes more like native HA (Campoccia et al., 1998). Once HYAFF® 11 is in contact with a wound, it integrates and begins to release HA. HYAFF® 11 fibers provide a sustained release of HA that promotes epidermal cell migration and new dermal tissue reconstruction (Price et al., 2007). Hyalomatrix® is a bi-layered, flexible, and sterile transparent membrane composed of HYAFF® 11. A thin and transparent silicone membrane comprises the upper layer, and a 3D fibrous matrix of HYAFF® 11 comprises the lower. The thin silicone layer keeps the wound moist, protects it, and monitors its healing process without removing the dressing. Moreover, the thin silicone layer is easily removed after the neo-tissue formation and does not damage the wound bed or cause pain (Longinotti, 2014). Hyalosafe®, which was also designed using HYAFF® 11 technology, is a transparent film indicated for the management of moderately superficial exuding wounds. This material does not cause pain because it does not adhere to the wound. As an example of its aesthetic results, Hyalosafe® was used on epidermal and superficial dermal burn wounds to protect the area from infections, and the wounds recovered without scarring (Merone et al., 2001). Hyiodine® is a complex composed of 1.5% HA sodium salt, 0.1% iodine, and 0.15% potassium iodide (Cutting, 2011; Vasir et al., 2003; Paghetti et al., 2009). The low concentration of iodine (0.1%) may reduce irritation and maintain antibacterial properties against hyaluronan-degradation bacteria. Hyalofill® is another cream-colored non-adherent dressing manufactured from HYAFF (Colletta et al., 2003; Khelfi, 2018). Hyalofill®-F interacts with wound exudates to produce a hydrophilic gel. Colletta et al. (2003) found that Hyalofill®-F had a beneficial effect even in treating difficult-to-heal venous ulcers. HylaSponge® is composed of spheroidal particles of nearly infinite MW that contain very large coils produced from a network of large HA molecules (Mahedia et al., 2016). These spheroidal particles with various MW HAs and potentially bioactive factors provide a “waterway” for absorbing and releasing abundant water. The high- or low-MW HAs and bioactive factors diffuse into the stratum corneum of the skin, while the low-MW HAs and bioactive molecules diffuse toward the skin’s deeper layers. Laserskin® is an HA sheet that is maximally esterified and has perforations 40–500 μm in diameter (Price et al., 2007). Harris et al. (1998) demonstrated *in vitro* that keratinocytes proliferate across and migrate through perforations to the wound bed. Laserskin® has been mainly studied in chronic and burn wound therapy, with the treatment of diabetic ulcers (Lobmann et al., 2003) and chronic leg ulcers (Hollander et al., 1999) showing high rates of healing.

**TABLE 2:** Manufacturers and characteristics of commercial HA-based wound dressings.

Product	Manufacturer	Characteristics
Hyiodine®	H&R Healthcare Ltd., Hull, United Kingdom	Mixture of HA and iodine
Bionect®	Fidia Farmaceutici S.p.A., Abano Terme, Italy	Topical solution composed of HA sodium salt (0.2%) that prevents abrasions, removes harmful agents, and restores skin integrity
Connettivina®	Fidia Farmaceutici S.p.A., Abano Terme, Italy	Cream composed of HA sodium salt (10 mg/mL) that provides a moist environment for promoting cell migration and skin regeneration
Hyalofill®	ER Squibb and Son, United Kingdom	Cream-colored non-adherent product of composed of HYAFF that provides a moist environment and can be used on chronic wounds; available as a flat sheet (Hyalofill-F) or rope (Hyalofill-R)
Hyalomatrix®	Fidia Advanced Biopolymers, Abano Terme, Italy	Transparent membrane composed of HYAFF that promotes capillary growth and cellular invasion processes
Hyalosafe®	Anika Therapeutics s.r.l., Abano Terme, Italy	Transparent film composed of HYAFF that covers superficial wounds and provides a moist healing environment
Hylase wound gel®	Sanofi Aventis, Paris, France	Gel composed of HA sodium salt (2.5%) and emollients that prevents tissue dehydration and improves wound healing
HylaSponge®	Matrix Biological Institute, Fort Lee, N.J., United States	Sponge composed of a network of large HA molecular chains that prevents a moist environment by absorbing and releasing large volumes of water
Laserskin®	Cynosure Inc., United States	Scaffold composed of HYAFF that supports the growth and migration of fibroblasts and autologous keratinocytes to the wound
HYAFF® 11	Fidia Farmaceutici S.p.A., Abano Terme, Italy	Derivative of HA produced by the esterification of HA with benzyl alcohol

## 5 Different types of wound dressings

### 5.1 Hydrogels

**FIGURE 4:**
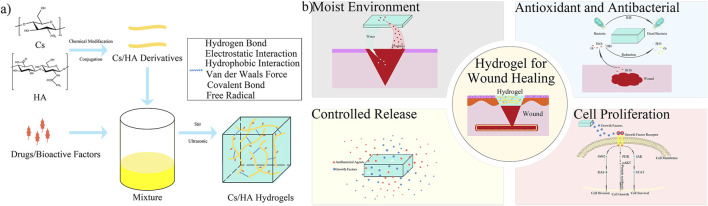
**(a)** Preparation process of CS- and HA-derivative hydrogel, loaded with drugs and bioactive factors, and crosslinking mechanisms of hydrogel, including hydrogen bond, electrostatic interaction, hydrophobic interaction, Van der Waals force, covalent bond, and free radical. **(b)** Bioactivities of hydrogel wound dressing, including maintaining a moist wound area, controlled release of drugs and bioactive factors, antioxidant and antibacterial effects, and facilitation of cell proliferation effects.

A moderately moist wound environment is important for effectively preventing tissue dehydration, cell death, and increasing EGF levels (Chen et al., 2018). Moreover, epithelial cells and keratinocytes rapidly migrate in a moist environment; these cells, in turn, affect wound epithelialization (Xu et al., 2020). Thus, it is essential for wound dressings to provide a moderately moist environment. Hydrogels produced from hydrophilic polysaccharides with a 3D mesh structure were found to quickly dissolve in water and form a semi-solid state containing more than 90% water (Yang X. et al., 2022). Hydrogels also retain water, which ensures a moderately moist wound environment and allows for wound exudates to be absorbed (Li A. et al., 2024). Hydrogels have been developed that facilitate the process of wound healing by removing necrotic tissue from wounds (Li S. L. et al., 2024). In addition, the distinct characteristics of hydrogels, including good mechanical strength, biodegradability, viscoelasticity, non-adhesion, and similarity to tissue (Tyeb et al., 2018), support its use in wound dressings, biomedicine, tissue engineering, and other applications (Mondal et al., 2024; Yan et al., 2024; Viola et al., 2024). Hydrogels have been categorized based on their crosslinking mechanism into two primary types: physically and chemically crosslinked gels (Pellá et al., 2018; Shariatinia and Jalali, 2018) (Table 3). Physical crosslinking occurs through hydrogen bonds, electrostatic interactions, hydrophobic interactions, and Van der Waals forces. pH-responsive hydrogels can be prepared because the carboxyl and amino groups have different forms at different pH values, leading to changes in the hydrogel’s ionic strength (Ma et al., 2024). Environmentally responsive and self-recovering hydrogels comprising two polymers (CS and sodium alginate) were synthesized, which showed excellent antibacterial properties (Ren et al., 2016). Although environmentally responsive hydrogel dressings have shown excellent control of drug release and can promote wound healing, traditional hydrogel dressings are still widely used in wound care. Several environmentally responsive hydrogel dressings are still in the preclinical stage. Wu et al. (2022) designed a pH/ROS stimuli-responsive hydrogel dressing that significantly promoted wound healing. In addition, long-term biosafety results revealed that pH/ROS stimuli-responsive hydrogel did not induce inflammation, an immune response, or necrosis, and the hydrogel completely degraded within 21 days. Chemical crosslinker can be used to synthesize hydrogels through chemical reactions to form covalent bonds or macromolecular polymers (Ma et al., 2024). However, common chemical crosslinkers, such as 1,4-butanediol diglycidyl ether and poly (ethylene glycol) diglycidyl ether, exhibit cytotoxicity (Jeong et al., 2021). Their cytotoxicity significantly decreased the viability of HaCaT and human dermal fibroblast (HDF) cell lines, altered the integrity and shape of the cell membranes, and caused high expression of COX-2 and inflammatory cytokines (TNF-α and IL-1 β). Therefore, chemical crosslinkers must be eliminated from hydrogels by dialysis or other methods. The Schiff base reaction, a reaction between an aldehyde group and an amino group, can form hydrogels with only one byproduct (water) (Suo et al., 2021). In addition, the free-radical crosslinking method was reported to avoid the need to remove chemical crosslinker. The Diels–Alder reaction used for generating hydrogels does not require a catalyst (Wu et al., 2025). Two other normally free-radical polymerization reactions, the oxime reaction (Grover et al., 2012) and the thiol-Michael addition reaction (Jin et al., 2010), are used to prepare hydrogel. UV-initiated free radicals can also polymerize without a catalyst. Wang et al. (2025) prepared CS–polyurethane hydrogels using bromocresol green and thiol-modified CS polyurethane grafted with eugenol for extending the shelf life of food through a UV-curing method. Natural polymer hydrogels showed better biocompatibility and biodegradability than synthetic polymer hydrogels and, therefore, have greater application potential in wound dressings (Sanyal et al., 2024). The preparation and applications of hydrogels for wound dressings are shown in Figure 4.

**TABLE 3:** Mechanism, composition, function, and wound model of different types of crosslinking hydrogel wound dressings.

Type	Mechanism	Composition	Function	Wound model	Reference
Physical crosslink	Hydrogen bond	C6-carboxymethyl-D-glucosamine, N-acetyl-D-glucosamine (β-1,4- Linked); N-vinyl-2 -pyrrolidone; 1,2,3,4,6-penta-O-galloyl-β-D-glucose	Promotes angiogenesis and granulation tissue	Total skin defect wound	Xiong et al. (2024)
	Electrostatic interaction	D-glucosamine, N-acetyl-D-glucosamine (β-1,4-linked); β-D-mannuronic acid sodium and α-L-guluronic acid sodium (1,4-linked)	Antibacterial effects	Infected wound	Ren et al. (2016)
	Hydrophobic interaction	D-glucuronic acid and N-acetyl glucosamine (β-1,3- linked); adipic dihydrazide, organoselenium polymer	Enhances angiogenesis	Diabetic wound	Zhao et al. (2026)
	Van der Waals forces	Malva nut polysaccharides; D-Glucosamine, N-acetyl-D-glucosamine (β-1,4-linked)	Supports keratinocyte migration	Total skin defect wound	Sun et al. (2025)
Chemical crosslink	Chemical crosslinker	D-glucuronic acid and N-acetyl glucosamine (β-1,3-linked); zinc oxide	Hemostatic and antibacterial effects	Infected wound	Rao et al. (2019)
	Schiff base reaction	D-glucuronic acid and N-acetyl glucosamine (β-1,3- linked); antimicrobial peptide	Antibacterial property	Chronic infected wound	Suo et al. (2021)
	Free radical Diels–Alder reaction	D-glucuronic acid and N-acetyl glucosamine (β-1,3-linked)	Enhances angiogenesis	Total skin defect wound	Wu et al. (2025)
	Free-radical oxime reaction	Eight-armed aminooxypoly (ethylene glycol)	Biocompatibility	​	Grover et al. (2012)
	Free-radical thiol Michael addition reaction	D-glucuronic acid and N-acetyl glucosamine (β-1,3- linked); poly(ethylene glycol)	Biodegradable property	​	Jin et al. (2010)
	UV-initiated free-radical reaction	D-glucosamine, N-acetyl-D-glucosamine (β-1,4-linked)	Antibacterial property	​	Wang et al. (2025)

#### 5.1.1 CS-based hydrogels

Based on amino and hydroxyl groups, CS is conducive for forming chemically crosslinked hydrogels with many types of polymer. CS physically crosslinked hydrogels have been formed based on electrostatic interactions, negatively charged molecules, and anions interacting with the amino groups of CS (Zhao J. et al., 2023). Khorasani et al. (2019) prepared polyvinyl (alcohol)/CS/nano zinc oxide hydrogels using the freeze–thaw method. The hydrogel pore size was 13.7 ± 5.9 μm on average, and water absorption was in the range of 680%–850%. An increased elastic modulus and tensile strength and decreased elongation at break resulted when the freeze–thaw cycles were increased and the thawing temperature reduced. The hydrogels showed greater antibacterial ability against *S. aureus* than *E. coli*. Cell viability and proliferation tests showed that the hydrogels were non-toxic to L-929 and HDF cells. Masood et al. (2019) formulated a CS–PEG hydrogel loaded with silver nanoparticles for wound healing. The hydrogel was highly porous with 72.2% ± 0.2% porosity and had a higher swelling capacity and water vapor transition rate (2,104 g m^−2^/24 h) than the bare CS–PEG hydrogel. The hydrogel showed improved antioxidant and antibacterial properties compared with bare CS–PEG hydrogel *in vitro* and continuously released AgNPs over 7 days. The hydrogel demonstrated good wound healing in diabetes-induced rabbits.

Modified CS with good water solubility has been shown to confer better qualities to hydrogels than raw CS. An et al. (2024) designed a multifunctional hydrogel wound dressing composed of CS modified with 4-carboxyphenylboronic acid and rutin. The hydrogel exhibited good biocompatibility with L929 cells treated with the hydrogel, maintaining a viability of over 97%, and exhibited potent free-radical scavenging activity, with scavenging ratios ranging from 53% to 70%. In addition, the hydrogel showed good anti-inflammatory effects and robust antimicrobial properties. It also accelerated wound healing, achieving 98% healing by day 10. Guo et al. (2024) designed self-healing and self-adhesive hydrogel dressings from adamantane-modified silk fibroin, *β*-cyclodextrin-modified filipin protein, and quaternary ammonium-modified CS. The host–guest interactions between *β*-cyclodextrin-modified filipin protein and adamantane-modified silk fibroin and the hydrogen bonding from quaternary ammonium CS provided the crosslinking mechanism. The hydrogels exhibited exceptional antibacterial characteristics against *S. aureus* and *E. coli*. Hydrogels incubated with NIH-3T3 cells showed excellent cytocompatibility and accelerated wound healing potential. Xue et al. (2019) formulated a quaternized CS–Matrigel–polyacrylamide hydrogel with a 3D microporous structure. The equilibrium swelling ratio of the hydrogel was approximately 1,200%. It showed excellent extensibility, compression, and resilience and had a strong adhesive force (0.52 N, similar to commercial wound dressings of 0.7 N). The hydrogel also showed good antibacterial and hemostatic properties, enhanced collagen deposition, facilitated anti-inflammatory factors, and accelerated wound healing. Li A. et al. (2024) prepared CS–polyvinyl alcohol hydrogels embedded with Ag-doped TiO_2_ nanoparticles for enhancing sonodynamic antibacterial therapy. The added Ag increased the hydrogels’ generated ^1^O_2_ signals, and ROS accumulated in the bacteria, seriously affecting the fluidity and permeability of the bacterial cytomembrane and killing the bacteria. Wang C. et al. (2024) constructed an ultrasound-responsive antibacterial hydrogel consisting of Fe_3_O_4_-grafted berberine, L-arginine-grafted CS, and poly(vinyl alcohol). The heterojunction formed between Fe_3_O_4_ and berberine increased the generation of ROS, which catalyzed the L-arginine grafted to the hydrogel to release nitric oxide, inhibiting the growth of bacteria and positively affecting the polarization of M1 macrophages. In addition, the berberine released from the hydrogels showed excellent anti-inflammatory effects and antioxidative activity.

#### 5.1.2 HA-based hydrogels

HA is a natural hydrophilic polymer with active functional groups that are easy to modify, exceptional biocompatibility, and have strong mechanical properties (Yang Y. et al., 2022), which are conducive to its wide application in wound dressings. Cai et al. (2024) constructed a multifunctional hydrogel comprising a dynamically crosslinked network doped with the polydopamine-modified zeolite imidazole framework-8, oxidized HA, and hydrazide-modified HA. The hydrogel enabled rapid hemostasis and showed excellent antioxidant activity. After culturing with the HPZ8 and being irradiated with 808 nm light, only 2.9% of *E. coli* and 10.3% of *S. aureus* survived. The Zn ions released from the hydrogel promoted cell migration and angiogenesis. In addition, the Zn ions accelerated the M2 polarization of macrophages to promote healing of the infected wound. Ferreres et al. (2024) synthesized a thiolated HA hydrogel that incorporated metal phenolic network nanoparticles composed of epigallocatechin gallate and cobalt. The metal phenolic network nanoparticles generated ROS, which disrupted bacterial membranes and reduced the survival rates of *S. aureus* and *P*. *aeruginosa*. In a wound microenvironment, the hydrogel reduced deleterious enzyme activity (inhibiting matrix metalloproteinases and myeloperoxidase by 60% and 80%, respectively), which promoted wound healing and controlled sustained oxidative stress, thereby improving cell proliferation and tissue remodeling. Exploiting the dynamic changes in pH that affect the clearance efficiency of ROS, Zhai et al. (2024) prepared a nano-composite hydrogel comprising ZnO nanoparticles and *O*-nitrobenzene-modified photo-triggered HA as a dressing for infected diabetic wounds. The hydrogel showed high antioxidant capacity and scavenged 54.57% and 60.1% of ROS at pH 5 and 7, respectively. The hydrogel also effectively inhibited bacteria, with antibacterial rates against *S. aureus* and *E. coli* of 64.03% and 58.35% after 6 h, respectively. In addition, *in vitro* tests showed that the hydrogel was biocompatible and enhanced angiogenesis and the formation of hair follicles.

HA is easily degraded by oxidants and enzymes *in vivo*. Many approaches have been used to circumvent this weakness, such as chemical modifications and functional substance loading and grafting (Raina et al., 2022). Zhao et al. (2020) constructed a photo-responsive hydrogel consisting of azobenzene, *β*-cyclodextrin groups, and HA. Azobenzene and *β*-cyclodextrin were conjugated to HA chains, and EGF was loaded in the hydrogel to promote wound healing. Leveraging the photoisomerization properties of azobenzene, azobenzene, and *β*-cyclodextrin assembled under visible light but dissociated under UV light caused EGF to be released from the loosened hydrogel under UV light. Therefore, the hydrogel conveniently delivered EGF to the wound site. EGF showed superior wound healing efficiency by accelerating granulation tissue formation and angiogenesis. Yang et al. (2021) developed bionic ECM hydrogels composed of oxidized HA and thiol-modified poly (*γ*-glutamic acid). The reaction between the -SH groups in poly(*γ*-glutamic acid) and -CHO groups in oxidized HA provided the hydrogel with biocompatibility and self-healing capabilities. The hydrogel exhibited *in vitro* and *in vivo* free radical scavenging, viscoelastic properties, and biodegradability similar to those of the natural ECM. Wound healing tests using a full-thickness skin defect model showed that the hydrogel enhanced angiogenesis and collagen deposition more than the commercial dressing Tegaderm™. Zhao Y. et al. (2023) prepared a hydrogel using glutaraldehyde crosslinked HA and polyacrylamide to form interpenetrating polymer networks to improve rapid wound contraction. The thermal response characteristics of the polyacrylamide caused the hydrophilic hydrogel to become hydrophobic when the temperature exceeded the lower critical solution temperature. Therefore, the hydrogel shrank after being placed on the wound site. The hydrogel also exhibited good tissue adherence. *In vivo* mouse model tests showed that the hydrogel achieved sutureless post-wound closure and accelerated wound healing through its anti-inflammatory properties, promoted angiogenesis, and reduced collagen deposition. The hydrogel also demonstrated good hemocompatibility and cytocompatibility.

#### 5.1.3 Challenges associated with hydrogel wound dressings

Hydrogels prepared with CS and HA have shown good biocompatibility with accelerated accelerating wound healing and are both promising wound dressings. However, there are also a number of challenges that they must overcome. Water-insoluble CS is usually physically crosslinking and has chemical modifications or conjugation. Physical or chemical methods are also needed to avoid HA by oxidants and enzymes *in vivo*. These physical intermolecular forces are generally weaker than chemical covalent bonds and are easily affected by the surrounding microenvironment, including temperature, pH, and medium composition. Hydrogen bonds increase gelatinization and decrease crosslinking density. Therefore, hydrogen-bond-crosslinked hydrogels have certain drawbacks, such as poor injectability and weak mechanical strength (Gupta et al., 2006). The dynamic covalent bonds used in chemically crosslinked hydrogels have shown promise for constructing smart responsive systems and multifunctional designs, but their clinical translation has been limited (Gao et al., 2025). Dynamic covalent bonds can be broken in extreme environments (e.g., strong acids and bases). Therefore, the stability of hydrogels should be optimized. In addition, chemical crosslinking moieties, such as aldehyde groups and boronic acid groups, are usually used to create dynamic covalent bonds. The agents remaining in hydrogels are commonly toxic and may cause cytotoxicity or allergic reactions. Therefore, the biocompatibility of hydrogels must also be optimized. Furthermore, the degradation of dynamic covalent bonds can lead to hydrogel over-swelling in wounds, inhibiting wound healing. Therefore, an appropriate degradation rate is another crucial property of wound dressing hydrogels. Several additional agents, such as silver and ZnO, have shown excellent antibacterial activity in hydrogels. However, excessive additional agents will inevitably increase cytotoxicity. Hence, it is crucial to embed appropriate concentrations of antibacterial agents.

### 5.2 Nanofibers

Nanotechnology, the fastest emerging miniaturized technology, is widely used in biomedicine, including drug delivery (Ambekar and Kandasubramanian, 2019a), skin tissue engineering (Ambekar and Kandasubramanian, 2019b), bone tissue engineering (Chiu et al., 2016), antimicrobial applications (Balasubramanian et al., 2015), and bio-adhesives (Korde and Kandasubramanian, 2018). Nanofibrous membranes, a relatively new wound dressing composed of many intersecting nanofibers, have a structure that mimics the ECM (Abrigo et al., 2014) and have demonstrated many positive qualities. Nanofibers facilitate optimal interaction between cells (e.g., fibroblasts and keratinocytes), have a large surface area which is beneficial for surface functionalization (Shariatzadeh et al., 2025), and provide a large cell attachment area for enhancing wound healing. Nanofibers also exhibit reticulated nano-porosity, which can efficiently inhibit microorganism survival and enhance hemostasis. Due to their exceptional porosity (ranging from 60% to 90%), nanofibers efficiently permeate substantial water and oxygen and exchange nutrients and metabolic waste (Andreu et al., 2015). In addition, the diameter of nanofibers can be precisely controlled to match that of skin protein nanofibers (ranging from 60 to 120 nm), which is essential for cell–scaffold interactions (e.g., adhesion, migration, and differentiation) (Young et al., 2016). Furthermore, nanofibers have shown outstanding compatibilities when combined with various agents and solvents, which has enabled bioactive factors (signaling factors and growth factors) to functionalize nanofibers, thereby improving their regenerative potential (Bombin et al., 2020). Nanofibers are fabricated using a variety of techniques (Table 4). Electrospinning is a widely used technique with high efficiency, simplicity, low production costs, and high adaptability and, therefore, is the most common technique used to fabricate nanofibers (Nune et al., 2017). Electrospinning apparatuses are usually based on uniaxial or coaxial electrospinning; they have only one nozzle, and a polymer solution with the desired drug–polymer ratio is extruded from a syringe tube column after volatilizing the solvents (Goyal et al., 2016). A polymer solution incorporating drugs and a polymer matrix can be prepared by chemical or physical methods. Nanofibers with a controlled drug release rate were developed by chemically linking ciprofloxacin and polylactic acid, with diameters of 150–400 nm and pore sizes of 62–102 nm (Parwe et al., 2014). A drug can also be physically incorporated into the polymer matrix through simple mixing. Based on this method, Zhang et al. (2014) synthesized vancomycin-coated titanium implants to inhibit implant-related infections. *In vitro* and *in vivo* tests have revealed that nanofibers have satisfactory antibacterial activity. To control nanofiber size, the main influencing factors have been explored and may include solution feed rate, charge strength, and solution conductivity. Under an electrical field, Weng and Xie (2015) showed the fiber diameter to be positively proportional to the solution feed rate, with the nanofibers becoming thinner as charge strength increases. The fiber diameter was also thicker when solution conductivity was higher (Hayati et al., 1987). A coaxial electrospinning apparatus with two separate and concentric spinnerets formed nanofibers with a core-shell architecture (Liu et al., 2019). Active components have also been incorporated into polymer matrices by physical and chemical linkage and are commonly located in the inner layer to avoid degradation by moisture and oxygen. Yang et al. (2017) designed a nanofiber with a core layer containing fish oil and Zn and a shell layer of polyvinylpyrrolidone (PVP). The fish oil, protected by the shell layer, showed significantly enhanced oxidative stability compared to single-layer nanofibers. The nanofiber enabled the sustained release of most of the encapsulated fish oil, which efficiently enhanced its bioactivity. Coaxial electrospinning has also been used to synthesize stimuli-responsive nanofibers. Han et al. (2017) developed a self-immolative polymer nanofiber for controlled release. The active substance was encapsulated in the smart shell layer, which quickly depolymerized in response to stimulating signals, rapidly releasing the active substance from the core layer. The active substance was not released from the shell layer without the stimulating signals. These examples demonstrate the stimuli-responsive abilities of nanofibers. Although coaxial electrospun nanofibers have shown excellent bioactivity, a stable interaction between the core and shell layers is one of the greatest challenges (Zhu et al., 2016). For example, separation of the two layers may lead to the loss of bioactivity. Their interfacial strength can be lowered by dissolving the polymer with similar solvents and adjusting their concentrations. In melt blowing, the melted polymer is extruded from dies by fast-flowing heated air, which deposits the polymer nanofibers onto a collection device (Tahalyani et al., 2018; Rogalski et al., 2017). However, the extremely high temperature used to melt polymers can cause degradation, which limits the use of melt blowing to produce nanofibers. Self-assembly is a method in which smaller molecular components converge into larger, defined molecular materials, such as nanofibers. Molecular interaction, including hydrogen bonds, Van der Waals forces, and weak covalent bonds drive the self-assembly process. To improve the interactions between components, a combination of interaction forces can be used (Xu et al., 2018). However, self-assembly has certain drawbacks, such as complex manufacturing processes and low production rates (Hedegaard and Mata, 2020). Thus, self-assembly has limited utility for producing nanofibers. Rotary jet spinning is another method used to produce nanofibers. A polymer is dissolved in a solution or melted into a liquid and then spun at a high angular velocity to produce jet expulsion though a nozzle. The rotated polymer solution forms nanofibers that are collected onto a collector (Rogalski et al., 2017). However, the use of rotary jet spinning to produce nanofibers is also limited due to the high temperatures required. Using a phase separation method, a polymer was dissolved in tetrahydrofuran, then treated using nonsolvent or thermal energy to separate the system into two phases (the upper phase was polymer and the lower phase was solvent) based on differences in physical properties, thereby causing gelation (Hedegaard and Mata, 2020). After this, the gel was freeze-dried, which removed the solvent. Finally, lyophilization yielded polymer nanofibers. The porosity and size of the nanofibers can be modified by changing the temperature or concentration of the polymer solution. However, the limits of this method, including limited polymer quantities, the inability to scale up, and time-consuming procedures, have limited its use in fabricating nanofibers (Chen and Liu, 2015). Hand spinning is a technology in which a viscous material is pulled manually with the thumb and index finger (Zahmatkeshan et al., 2019; Lee et al., 2016). Using this technology, highly concentrated and well-aligned nanofibers were generated that were unaffected by the electrical properties of the polymer or solvent; however, throughput was low. Template synthesis is widely used to fabricate nanomaterials (Zahmatkeshan et al., 2019). In this method, a template is used to extrude nanofibers. The template (commonly an aluminum oxide membrane with many holes) can be modified to adjust the porosity and size of the nanofibers. However, post-synthesis template removal is difficult, limiting its application. The use of pressurized gyration is also limited, in this case by excessively high temperatures (Heseltine et al., 2019). Another technique, drawing, was used to form nanofibers by contacting a glass rod or micropipette with a millimetric droplet of a polymer solution on a SiO_2_ surface and slowly drawing away to form nanofibers (Almetwally et al., 2017). However, the diameters of the nanofibers were reported to be 100 nm or greater, which limits its application. Centrifugal spinning is a technique in which a polymer solution is ejected from a spinneret containing two or more orifices under the effect of centrifugal force, with the nanofibers being deposited on the collector (Stojanovska et al., 2016). However, the nanofibers, thus, formed can be difficult to collect.

**TABLE 4:** Advantages and disadvantages of different nanofiber fabrication methods.

Method	Advantage	Disadvantage
Electrospinning	Fiber size ranges from a few microns to a few nanometers; high aspect ratio; low-cost technology; enhanced mechanical characteristics	Instability of jet; requirement of toxic compounds; finite control of pore size
Melt blowing	High yield; long fibers; no solvent recovery	Instability and limited polymer types
Self-assembly	Used to generate multifunctional nanofibers	Complex process; expensive; low yield
Rotary jet spinning	Eco-friendly; does not use high voltages	High temperatures
Phase separation	Controlled structure and pore size; minimal equipment requirement	Limited to specific polymers; short fibers
Hand spinning	High-quality and well-aligned nanofibers; high efficiency; independent on electrical properties	Low yield
Template synthesis	Different fiber diameters based on various templates	Template can be difficult to remove
Pressurized gyration	High yield; no electrical field; easy to generate homogeneous fibers and control product morphology	Very high temperatures
Drawing	Minimal equipment require	Minimum diameter of approximately 100 nm; non-continuous process
Centrifugal spinning	High yield; low cost	Difficult to collect nanofibers

#### 5.2.1 CS-based nanofibers

Electrospun CS nanofibers have marked advantages due to their specific surface area, porosity, and biological regeneration potential (Porrelli et al., 2021; da Mata et al., 2023). CS nanofibers are mainly used as antibacterial, hemostatic, angiogenesis, and immune regulation agents in wound healing (Cui et al., 2021). Ghasemian Lemraski et al. (2021) developed a double-layer electrospun nanofiber composed of a protective layer (including CS, polyvinyl alcohol, and copper) and a second layer (PVP). The results showed that the copper inhibited all tested bacteria, with greater inhibition of Gram-positive than of Gram-negative bacteria. Dai et al. (2019) synthesized CS and titanium dioxide (TiO_2_) microparticles incorporated on polylactic acid nanofibrous mats using electrospray technology. The TiO_2_ content of the electrospun membranes scattered by the CS/TiO_2_ microparticles was controlled by adjusting the concentration of TiO_2_. The 1.5 bilayer of the CS/TiO_2_ composites demonstrated the greatest antibacterial activity against *S. aureus* and *E. coli* (up to 95%) and the highest cell viability (about 92% and 95% on days 3 and 5, respectively). CS can function as a hemostatic agent because it forms cationic clusters that interact with anions on the RBC, causing RBCs and platelets to rapidly aggregate and stop blood loss (Nakielski and Pierini, 2019). Ren et al. (2019) prepared electrospun nanofibers blended with CS, silk fibroin, and halloysite nanotubes loaded with chlorhexidine digluconate. The halloysite nanotubes notably improved the tensile properties of the nanofiber, with blood clotting time decreasing from 13.29 min to 0.46 min when the halloysite nanotube content in the nanofibers increased. In addition, the release time of the drug increased by approximately 8 days after the halloysite nanotubes were incorporated into the nanofibers. Sasmal and Datta, (2019) prepared electrospun CS/poly(vinyl alcohol) nanofibers containing tranexamic acid. Hemostatic activity tests showed that the pure CS/poly(vinyl alcohol) nanofibrous membranes decreased blood clotting time from 210 ± 10 s to 167 ± 6 s as the concentration of CS increased, and tranexamic acid significantly reduced clotting time and plasma recalcification time. Angiogenesis is particularly important in skin tissue regeneration. Pal et al. (2017) reported a highly porous nano-/microfibrous scaffold using CS and polycaprolactone. The results of a histological examination showed that a provisional matrix was formed by host cellular and blood vessel infiltration by day 3 post-wounding. The results revealed that the CS/polycaprolactone scaffold effectively improved angiogenesis. The inflammation stage is necessary for wound healing, but chronic inflammation restricts its progression. Therefore, appropriate immune regulation is crucial for treating chronic wounds. Zanchetta et al. (2020) evaluated the effect of polycaprolactone coated with CS/poly(ethylene oxide) electrospun fibrous membranes on mouse wounds. Wounds treated with the fibrous membranes showed abundant inflammatory infiltrate and higher levels of tumor necrosis factor-α and cell nuclear antigen than those treated with polycaprolactone membranes only. Thus, CS/poly(ethylene oxide) provided a level of immune regulation.

#### 5.2.2 HA-based nanofibers

Nanofibers have been constructed from natural and synthetic polymers (e.g., HA) using electrospinning (Astaneh and Fereydouni, 2023). HA has primarily been used for its angiogenesis and immune regulation effects during wound healing. Xing et al. (2023) artfully designed a nanofibrous hydrogel formed by crosslinking HA with L-phenylalanine and cationic hexapeptide co-assembled nanofibers though hydrogen bonding. The nanofibrous hydrogel had abundant cationic sites and chirality that interacted with advanced glycation end-products in a stereoselective manner, reducing their concentration. The nanofibers also increased vascular endothelial growth factor. The nanofibrous hydrogel showed remarkable angiogenesis and re-epithelialization activity; they also shortened the wound healing from 21 to 14 days. Liu et al. (2020) synthesized electrospun thioether-grafted HA nanofibrous hydrogels that modulated the inflammatory microenvironment of chronic wounds. The thioethers quickly scavenged ROS to attenuate inflammatory reactions, and the nanofibrous hydrogels increased the transformation of M1 macrophages to the M2 phenotype. The diabetic wound area treated with the nanofibrous hydrogels was smaller than that treated with nanofibrous hydrogels without grafted thioethers.

#### 5.2.3 Challenges associated with nanofiber wound dressings

Although polysaccharide-based nanofibers have shown tremendous potential for use in wound healing, some drawbacks still must be addressed for their clinical application. The main drawback of nanofibers is difficulty in controlling their physicochemical characteristics during the preparation process (Priya et al., 2022). Electrospun fibrous membranes have shown weak mechanical stability. Their production rate is low, and thus, it is difficult to manufacture electrospun fibrous membranes on a large scale (Su et al., 2024). Therefore, new studies and technologies are crucial to accurately control the physicochemical characteristics of nanofibers and increase their output. Strategies for enhancing the mechanical properties of nanofibers have been described in numerous studies (Salleh et al., 2025). Methods for improving the mechanical properties of electrospun nanofibers can be divided into structural and material modifications. Structural modifications include altering fiber orientation, core-shell structures, and nanofiber layering. In the preparation of nanofibers, the hydrogen bonds that are formed hinder effective chain alignment. To enhance the mechanical properties of a nanofiber, Zhang et al. (2026) fabricated high-performance anisotropic CS fibers using a sacrificial micelle-assisted alignment method. Firstly, sodium dodecyl sulfate was used to disrupt the hydrogen bonds through strong electrostatic interactions between the negatively charged sodium dodecyl sulfate and the protonated CS. Secondly, sodium dodecyl sulfate mitigated the electrostatic repulsion of CS, allowing it to slide and align. Finally, sodium hydroxide was added to remove the sodium dodecyl sulfate and reform hydrogen bonds. Material modifications include mixing with other polymers, the addition of fillers, and modification to the surface of the nanofibers using low-temperature plasma. Of these, low-temperature plasma surface modifications are particularly popular because they are non-destructive (Can-Herrera et al., 2016). Although developed, nanofiber wound dressings with appropriate mechanical stability require further research.

### 5.3 Films

Films are an important type of wound dressing. Gas can easily permeate a film while liquid and bacteria do not. Films are flexible, allowing them to easily conform to wounds in different body areas—even joints. In addition, films can be designed to allow some moisture evaporation, alleviate pain, and enable easy wound inspection (Savencu et al., 2021). Recently, polysaccharide-based films have been investigated as wound dressings (García et al., 2017). They have been shown to efficiently manage both acute and chronic wounds (Ke et al., 2020). With their excellent bioactivity, including swelling, tissue adhesion and biodegradability, films have been applied in the field of wound healing for hemostasis, wound monitoring, and antibacterial, anti-inflammatory, antioxidant, and stimulus-reactivity effects (Priya et al., 2024). Because of these properties, film wound dressings are widely used on superficial wounds due to their weak absorbency, on joint wounds due to their good flexibility, and on post-operative wounds due to their excellent waterproof characteristics and wound monitoring ability (Wu et al., 2024). Hydrogel and nanofiber wound dressings are applied to different types of wounds. The former have excellent moisturizing performance, analgesic effects, and fillability; therefore, hydrogel wound dressings are widely used for dry wounds, black necrotic wounds (e.g., pressure sores and diabetic foot ulcer), and minor burn wounds (Pan et al., 2025). Nanofiber wound dressings can promote cell proliferation and migration, transport drugs (e.g., antibacterial agents), absorb exudate, and reduce scar formation; they are thus widely used in acute, chronic (e.g., diabetic foot ulcer and venous ulcer), infected, and burn wounds (Shariatzadeh et al., 2025). Among such wound dressings, electrospun nanofiber dressings are ideal for chronic wound healing because nanofibers can incorporate and sustainably release various bioactive agents to assist chronic wound healing. Hydrogel wound dressings loaded with bioactive components have mainly been used in both non-infected and infected diabetic wounds, while the film wound dressings have mainly been used in superficial wounds (Tran et al., 2023).

**FIGURE 5:**
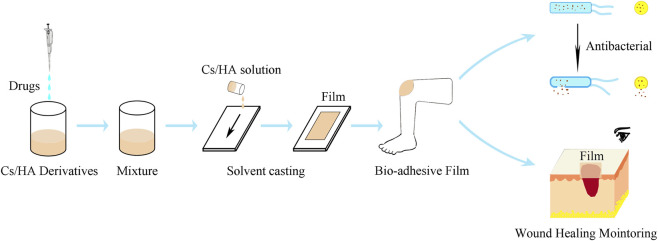
Preparation process of CS- and HA-based bio-adhesive film wound dressing loaded with drugs through solvent casting and its antibacterial property and application to monitoring wound healing.

Preparation methods are crucial to obtain wound dressing films with good flexibility, adhesion, resistance to tearing, ease of removal from packaging, exudate absorption, and active release (Savencu et al., 2021). The primary method used to prepare films is solvent casting because they can be produced using low-cost processing and facile manufacturing (Silvestro et al., 2020). In this method, the polymer and specific extracts or drug solutions are added to the solvent at defined temperatures (approximately 100–120 °C) and mechanically stirred until homogenous. The homogeneous blend solution maintains a constant volume and is poured into molds, where it is completely dried to obtain robust films. Another low-cost and -energy technique is salt, which is based on the insolubility of inorganic salts (e.g., sodium chloride) in typical organic solvents (Joerg et al., 2005). In one study, polymers were dissolved in a typical organic solvent, and the inorganic salts were added to the polymer solution. The homogeneous mixture was poured into Petri dishes and then washed with deionized water to remove the inorganic salt. Microfluidic spinning is an efficient method for producing micro- and nanoscale materials and is suitable for volatile compounds (Cheng et al., 2017). Based on microscale fluid dynamics principles, the core and sheath flows form a coaxial flow, and the coaxially flowing polymer dispersion is solidified by UV light, ionic, or chemical crosslinking and solvent exchange to obtain microfibers. These can be formed into a film using an immobilization device as a forward or reverse step in the process. This technology is suitable for preparing ultra-small-scale batches. In addition, 3D printing has been used to produce film scaffolds (Savencu et al., 2021) which allow over control their size, porosity, and shape; this is therefore appropriate for developing various films specific to individual requirements (Muwaffak et al., 2017). The preparation process and improving healing effects of film wound dressings are shown in Figure 5.

#### 5.3.1 CS-based films

CS has good bio-adhesive qualities and is also used in wound dressing films (Ueno et al., 2001). Li et al. (2022) synthesized guanidinylated CS films loaded with bioactive factors for wound healing. The SEM results revealed that nanoparticles had formed. The nanofilms were non-toxic to L929 cells, and wound healing results showed that CGNP1 exhibited styptic and analgesic effects, accelerated wound healing, and limited scar formation. Sezer et al. (2007) prepared a CS film containing fucoidan for burn healing. This showed high water vapor permeability (3.3–16.6/0.1 g), dramatically enhanced by freeze-drying, and excellent tensile strength (7.1–45.8 N), which increased with the concentration of CS. The film exhibited excellent bioactivity for skin regeneration, inducing re-epithelization and wound healing within 14 days. García et al. (2017) developed bioadhesive films containing ciprofloxacin-loaded CS crosslinked with PVP. The bioadhesive films loaded with ciprofloxacin showed high antibacterial activity against both Gram-positive and -negative bacteria. In addition, bioadhesive films increased the dressing’s residence time and reduced the number of administrations. Pereira dos Santos et al. (2019) prepared CS films that incorporated different concentrations of clove and melaleuca essential oil. All the CS films exhibited good transparency, flexibility, mechanical resistance, and high wettability and were thinner than the dermis. The films containing clove essential oil showed the greatest antibacterial activity.

#### 5.3.2 HA-based films

HA wound dressings exert anti-inflammatory effects during chronic wound healing (Priya et al., 2024). Li et al. (2018) prepared HA-grafted pullulan films to promote wound healing. The films had a leaf-shaped cascading arrangement with numerous small pores (about 73.13 μm) and therefore had a high swelling ratio. The films absorbed a significant amount of exudate, protecting the wound bed and reducing the frequency of replacement. Film composition was stable, with the complete degradation of HA-grafted pullulan films requiring 12–14 days. Thus, the films promoted wound healing. Abednejad et al. (2019) designed an HA/zeolite imidazolate framework nanoparticle film as a wound dressing with high stability and mechanical strength. The film exhibited a high Young’s modulus of 176 KPa and a low water contact angle of 27.7°, which confirmed its effective hydrophilicity. The film also demonstrated suitable adhesion and cell viability, with a released Zn ion level maintained within safe limits. Wang et al. (2022) fabricated CS- and HA-grafted pullulan succinate films using freeze-drying. The film had a 3D cavity structure and an excellent swelling ratio that absorbed a large volume of liquid to maintain a moist environment. It exhibited no cytotoxicity and had good antibacterial activity against *E. coli* and *S. aureus*. In addition, the film reduced inflammation and accelerated wound closure better than a marketed dressing.

#### 5.3.3 Challenges associated with film wound dressings

Polysaccharide-based films were developed primarily for their attractive mechanical abilities and efficiency when applied as wound dressings. However, research on bio-adhesive films has been stagnant due to numerous restrictions (e.g., cost, safety, and the fabrication process) (Sun et al., 2022). Several functional polysaccharide-based bio-adhesive films have been designed, but it has been difficult to adapt the dressings to the entire wound healing process (Yang and Wang, 2023). Another challenge has been controlling the biodegradation of bio-adhesive films for compatibility with the healing process. Notably, some bio-adhesive films loaded with electronic components have been designed that precisely and thoroughly indicate the wound healing process (Yang and Wang, 2023).

### 5.4 Sponges

Sponge particulates have many beneficial characteristics, including high porosity, large surface area, elastic deformation, and a 3D reaction environment (Zhang et al., 2021). The tiny porous structures of sponges provide ample reservoirs for a broad range of drugs to be shielded and shuttled. Therefore, sponge particulates are widely used in wound healing, drug delivery, and tissue engineering. The morphology of wound dressings is important for wound healing, particularly the pore structure. The pores of sponges typically have good connectivity, with the size of the pores in a sponge regulated by different production techniques and preparation methods. Sponges with appropriate pore sizes show excellent water permeability and nutrient transport. In addition, the pore structure of sponges can facilitate cell proliferation and their migration, vascularization, and tissue regeneration in wounds without the assistance of growth factor (Liu J. et al., 2023). Sponge dressings are the main conventional hemostatic dressing currently used, with the application of sponges dating to 1945 (Guo et al., 2021). Because of the high porosity of sponges (up to 97.9%), they can accumulate platelets and RBCs, concentrating coagulation factors to control hemorrhaging (Guo et al., 2021). Other wound dressings have pores and exhibit varying functions. For example, hydrogel wound dressings with a 3D mesh structure can contain more than 90% water. However, this structural feature can cause the hydrogel wound dressings to have weak O_2_ permeability. Macroporous hydrogel wound dressings have been designed to overcome this challenge and can also load various drugs (e.g., antibacterial agents and growth factors) for controlled release in the wound area. In addition, this pore structure has been found to facilitate cell migration to promote wound healing. However, the preparation of macroporous hydrogels is complex, and their fixed geometry is difficult to adapt to irregular wound areas (Kang et al., 2025). Nanofiber wound dressings mimic the ECM structure and have shown high porosity (60%–90%), facilitating water and oxygen permeation, protein absorption, and cellular migration to promote wound healing. To increase porosity and promote wound healing, nanofibers with a high pore size (2,911.9 nm) and porosity (92.6%) were prepared by aqueous phase fiber reassembly technology. When applied as a dressing, full wound closure was observed within 2 weeks (Yu et al., 2019). The interconnected porosity of nanofiber wound dressings was optimized to generate nanofibers that produced scar-free healing (Shariatzadeh et al., 2025). Film wound dressings with pores were designed to facilitate water and oxygen permeation while preventing bacteria permeation. The films produced efficient analgesic effects and were transparent. Omiderm®, a film wound dressing that simulates human skin, shows good flexibility, is non-stick, and is widely used for burns. In partial-thickness burn patients using a hydrophilic polyurethane membrane type of Omiderm®, re-epithelialization was complete in 8 days (Sahin et al., 2019). Scaffold wound dressings have been designed with 3D and porous structures. In wound care, the porosity of scaffolds is crucial for cell infiltration and angiogenesis activities because the interconnected pores transport nutrients and oxygen but prevent bacterial permeation. Natural scaffold materials (e.g., CS and HA) have shown good biocompatibility with scar-free healing. In the proliferation stage of wound healing, biodegradable scaffolds such as Permacol® and Lando® provide ideal growth conditions for new cells and tissue (Wang J. et al., 2024).

**FIGURE 6:**
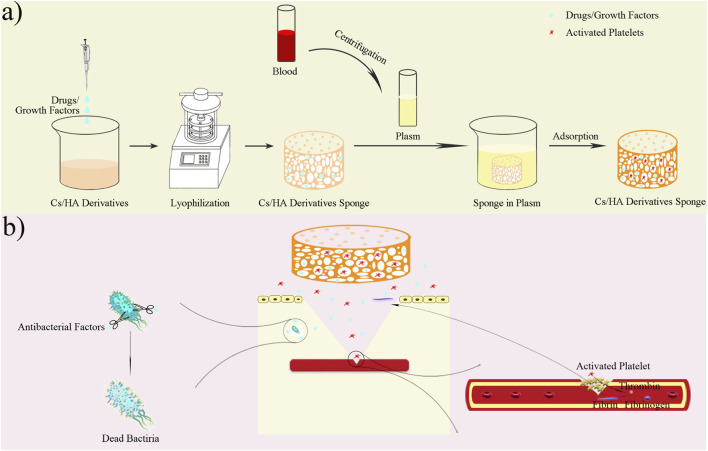
**(a)** Preparation process of CS and HA derivatives sponge loaded with drugs, growth factors, and activated platelets. **(b)** Bioactivities of sponge wound dressing, including antibacterial and hemostatic effects.

To control sponge morphology and enhance the diversity of loaded drugs, production techniques are continuously being optimized (Table 5). Current preparations of sponge particulates include traditional methods (double-emulsion solvent evaporation, liquid–liquid suspension polymerization, quasi-emulsion solvent diffusion, and oil-in-oil emulsion solvent diffusion) and novel methods (electro-hydrodynamic atomization and self-assembly). One commonly used method is double-emulsion solvent evaporation (Yang et al., 2000). An organic phase containing polymers and an aqueous phase containing emulsifiers is first mixed and stirred to form a primary emulsion. Second, a W/O/W emulsion is formed by dispersing the primary emulsion into a polyvinyl alcohol aqueous solution, often with ultrasonic or continuous agitation. Finally, the solvent is evaporated, and microsponges form under continuous agitation. One method suitable for loading inert nonpolar drugs is the liquid–liquid suspension polymerization method (Hainey et al., 1991). Monomers and drugs are dissolved in an organic phase, which is suspended in an aqueous solution with dissolved surfactants and dispersants. Crosslinking between monomers generates a polymer under stirring aided by a catalyst, increased temperature, or irradiation. Microsponges are obtained after filtration and drying. This polymerization process is quite long, and the loading rate of drugs is very slow. Another simple method is the quasi-emulsion solvent diffusion method (Jelvehgari et al., 2006). Polymers are dissolved in an organic phase, and a hydrophobic drug is gradually added while stirring. The organic phase is then dispersed into an aqueous phase containing polyvinyl alcohol and stirred to form a quasi-emulsion. The solubility of the drugs and polymers decreases as organic solvents evaporate and water infiltrates, forming porous microsponges. These are also prepared using oil-in-oil emulsion solvent diffusion (Abdellatif et al., 2018). Generally, acetone is used to dissolve polymer, drug, and Mg stearate, which is sonicated to obtain a homogenous dispersed phase. The mixture is dispersed into another oil phase (e.g., liquid paraffin) under gradual heating and stirring. Solidified microsponges are obtained by completely removing the acetone though diffusion and evaporation. Organic solvents, high temperatures, and irradiation are often used in conventional preparation methods, although these reaction conditions may destroy thermosensitive molecules and biological components (Zhang et al., 2021). Organic solvents such as methylene chloride used in the organic phase are hydrophobic, which reduces the solubility of hydrophilic drugs and bioactive factors, limiting their absorption in wound areas. In addition, the organic solvents are toxic to patients. Moreover, the high temperatures and irradiation used to prepare scaffolds are severe reaction conditions that can disrupt the structure of thermosensitive molecules and cause protein denaturation; thus, the drugs and bioactive factors may lose their potency after exposure to high temperatures and irradiation. Electro-hydrodynamic atomization technology has been used to quickly transform medium into droplets with proper diameters by adjusting their viscosity and voltage, forming monodisperse droplets that were freeze-dried to obtain microsponges (Pancholi et al., 2009). Another novel preparation method is nucleic acid self-assembly, which has been used to treat complex diseases (e.g., cancer and neurodegenerative disorders) (Evers et al., 2015). Linear single-stranded DNA containing specific sequences was designed with closed circular DNA formed at each end of the strand. The closed circular DNA was incubated with ribonucleotides and RNA polymerase for several hours at 37 °C to obtain long replicated RNA strands, which self-assembled to generate porous microsponges. The preparation process and improving wound healing effects of sponge wound dressings are shown in Figure 6.

**TABLE 5:** Advantages, disadvantages, and applications of different sponge fabrication methods.

Method	Advantage	Disadvantage	Application
Double-emulsion solvent evaporation	Prepared without heating; can be loaded with hydrophobic and hydrophilic bioactive agents	Persistence of surfactants in the sponges	Loads antibacterial agents, anti-inflammatory drugs, or anticancer medicines for wounds or cancer therapy
Liquid–liquid suspension polymerization	Potential to modify to a one-step method	Requires catalysis, high temperatures, or irradiation; long time; slow loading rate	Loads hypoglycemic or anti-inflammatory drugs for diabetic wound or inflamed tissue therapy
Quasi-emulsion solvent diffusion	Loads a substantial quantity of drugs; protects drugs; easy to control sponge size	Cannot be applied to hydrophilic drugs and polymers	Loads antibacterial agents, anti-inflammatory drugs, or anticancer medicines for fungal infection, arthritis, or cancer therapy; targeting and controlling release
Oil-in-oil emulsion solvent diffusion method	Loads a substantial quantity of drugs	Difficult to remove organic solvents	Loads hypoglycemic or anti-inflammatory drugs for diabetic wound or inflamed tissue therapy
Electro-hydrodynamic atomization	Efficient; loads both hydrophobic and hydrophilic drugs	High expertise required	Drug delivery
Self-assembly	Uniform size; low toxicity; mild reaction conditions	Expensive; only for gene	Gene delivery

#### 5.4.1 CS-based sponges

CS sponge dressings pose numerous advantages, including the absorption of fluids and the accelerated aggregation of RBCs and platelets; their compression properties also enabled them to effectively stop bleeding (Liu Z. et al., 2023). Shi et al. (2023) prepared expanding sponges with an interpenetrating network containing CS and plasma. The CS sponges were loaded with various active coagulation factors from the plasma to enable the treatment of wounds with high-pressure arterial bleeding. In this process, fibrin rapidly sealed and adhered to the wound, while thrombin catalyzed the formation of fibrin and promoted auto-coagulation. Other sponges with excellent hemostatic properties have also demonstrated antibacterial properties. Ren et al. (2023) synthesized a composite sponge composed of hydrophobically modified CS and gallic-acid-modified CS. The composite sponge rapidly stopped bleeding within 52 s, and its antibacterial effectiveness against *S. aureus* and *E. coli* reached 98.2% and 100%, respectively. The mechanisms of the antibacterial effects were also explored. The hydrophobic alkyl chains inserted into the cytomembrane of the bacteria and the polyphenol groups changed the permeability of cytomembrane and interacted with cell wall synthesis enzymes to promote cell death.

#### 5.4.2 HA-based sponges

HA-based sponge wound dressings are used clinically, such as the HylaSponge®. Sponges are non-adhesive and, therefore, usually require additional dressings, tapes, or bandages to maintain them at the wound site (Villamizar-Sarmiento et al., 2019). However, these additional dressing components may affect patient compliance and wound management. The tapes used to maintain sponge dressings may cause allergic reactions in patients with delicate skin due to the adhesive. Self-adhesive bandages without a separate adhesive are less likely to stay in place. Moreover, frequent wound dressing changes can result in skin avulsion, so it is crucial to maximize the residence time of dressings. However, there are numerous challenges associated with maximizing the residence time of sponge dressings, including displacement, excessive exudate, and perspiration (Amsler et al., 2009). Furthermore, additional dressings should be used in coordination with HA-based sponge dressings for efficient wound management. Catanzano et al. (2018) designed alginate and HA sponge dressings loaded with tranexamic acid. The sponge was produced using a straightforward internal gelation method with a freeze-drying step. The sponges were soft, flexible, porous, elegant, and nonbrittle. The tranexamic acid-loaded sponges provided controlled tranexamic acid release for up to 3 h and significantly reduced the blood clotting index by 30% compared with blank sponges. Yasuhiro Matsumoto and Kuroyanagi (2010) produced an HA-based sponge with arginine and EGF for wound healing. The sponge demonstrated good control over the inflammatory response. The levels of MPO produced by neutrophils were evaluated, revealing that arginine induced approximately 0.21 units/mg of MPO in the sponge containing arginine and EGF. The results of healing tests for wounds approximately 15 mm in diameter showed that the sponge promotes remarkable wound healing. Therefore, the synergic effect between HA, arginine, and epidermal growth factor promote wound closure and epithelization.

#### 5.4.3 Challenges associated with sponge wound dressings

Sponge dressings have shown great progress in the biomedicine field over the past decades, particularly in anti-infection and wound healing applications (Zhang et al., 2021). However, studies on fabrication techniques of sponge particulates with homogeneous dimensions and pore sizes are inadequate, confronting the clinical translation and production of sponges with major challenges (Zhao, 2013). To solve this issue, microfluidic technology and electrohydrodynamic atomization have been proposed as new manufacturing techniques (Arzi and Sosnik, 2018), while systematic research on the correlation between the internal pore size of sponges and production parameters is still needed (Zhang et al., 2021). At present, CS hemostatic sponges have been clinically used for wound care, while HA-based sponges have had limited clinical use as wound dressing. According to the *Medical Device Supervision and Administration Regulations*, HA-based medical dressings are limited to sodium hyaluronate hydrogel (classification number: 14-08-02), while HA-based sponges were determined to be a non-medical device. Supervision and administrative regulations improved in 2022; going forward, HA-based sponges will be more regulated. The sterilization of HA-based sponge dressings also limits their clinical translation because thermosensitive HA dressings can be sterilized by radiation, but radiation may degrade bioactive factors. In addition, functional HA-based sponge wound dressings are costly, further limiting their clinical translation.

### 5.5 Scaffolds

**FIGURE 7:**
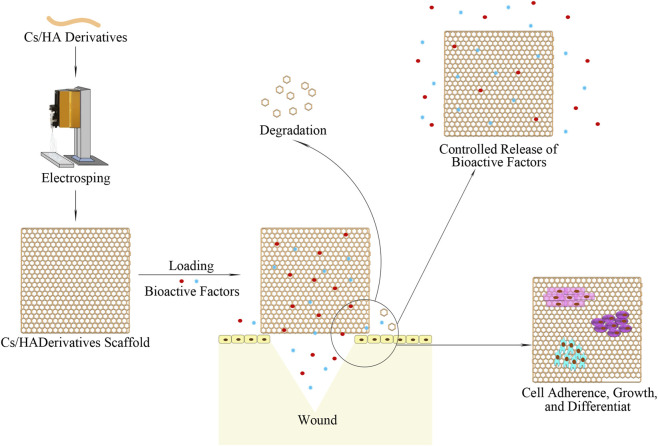
Bioactivities of CS- and HA-derivative scaffold wound dressing loaded with bioactive factors including good degradation and controlled release, facilitating cell adherence, growth, and differentiation.

Scaffolds composed of natural and synthetic polymers have been designed to treat infected wounds. They usually have a porous architecture and 3D structure (Zhong et al., 2010), which provide a substrate for cell adhesion, facilitate the proper exchange of nutrients and gases, and promote wound healing (Hsu et al., 2016). Effective wound healing by scaffolds is primarily based on the following principles as shown in Figure 7: (1) mimicking the ECM to provide an optimal niche for the adherence, growth, and differentiation of cells; (2) conforming to the wound bed shape and providing strong support; (3) facilitating cellular functions, including cell spreading, filling, and navigation within the scaffold; (4) allowing for the controlled release of bioactive agents to improve blood vessel formation and cell growth while minimizing infection risk; (5) enhancing functional integration through a degradation rate and mechanical strength tuned to the demand of the regenerating tissue (Anitha et al., 2025). Scaffolds are fabricated using techniques including electrospinning, freeze-drying, phase separation, solvent casting, fused deposition modeling, stereolithography, gas foaming, and inkjet printing (Anitha et al., 2025). The electrospinning, freeze-drying, phase separation, and solvent casting techniques are similar to those described in previous sections. Fused deposition modeling is a 3D printed technique for fabricating scaffolds (Mallikarjuna et al., 2025). A thermoplastic filament is placed into a heated extruder and melted in thin layers, then solidified to create scaffolds with the required geometry and porosity. The technology produces scaffolds with accurate geometrical structures and reproducibility (Narayan et al., 2021). Stereolithography is an advanced method for generating complex scaffolds (Hull, 1984). An ideal 3D image scaffold model is constructed and filled with a liquid photopolymer resin, polymerized and solidified by a UV light source layer by layer, and then cured and deblocked. Stereolithography can be used to produce scaffolds with high accuracy and surface quality, intricate structures, and unique designs. Porous scaffolds have been prepared using gas foaming, in which gas bubbles are introduced into a polymers to create a 3D porous structure (Bianchi et al., 2024). Therefore, this method can be used to control the size and distribution of pores in scaffolds. Inkjet printing is a complex technique for generating scaffolds (Derby et al., 2008). Infill polymers with specific rheological properties are deposited by a printer in the form of small droplets, which eventually form a scaffold. Inkjet printing can be used to develop scaffolds with personalized and complex patterns by customizing mechanical and biological properties.

#### 5.5.1 CS-based scaffolds

CS scaffolds combined with synthetic or natural polymers, oils, extracts, and drugs have been used as wound dressings (Ahmed and Ikram, 2016). Liu et al. (2017) fabricated CS-silk fibroin composite scaffolds loaded with Ag nanoparticles as a wound dressing. The scaffolds showed good moisture retention, high porosity, and appropriate tensile strength. *In vitro* antibacterial tests demonstrated that the scaffolds had good antimicrobial activity towards *P. aeruginosa*. Wounds treated with this scaffold healed within 14 days, demonstrating their utility as wound dressings. Bakhsheshi-Rad et al. (2020) prepared CS–alginate nanofiber scaffolds with different amounts of gentamicin *via* electrospinning. The scaffolds efficiently inhibited bacterial growth. The scaffolds with 1%–3% gentamicin demonstrated better attachment to L929 cells and promoted cell proliferation, and the scaffolds with 3% gentamicin promoted wound healing.

#### 5.5.2 HA-based scaffolds

HA-based scaffolds have high water absorption, which provides a moist environment for wound healing (Dovedytis et al., 2020). Alissa et al. (2025) prepared HA-based 3D scaffolds combined with hyperbaric oxygen therapy to promote diabetic wound healing. The treatment reduced neutrophil infiltration and pro-inflammatory cytokine levels and increased fibroblast proliferation, angiogenesis, and regenerative cytokine levels. Cai et al. (2025) produced an enzyme-crosslinked HA hydrogel to promote acute wound healing. The HA was grafted with dopamine, followed by enzyme crosslinking (horseradish peroxidase and hydrogen peroxide). The formed hydrogel scaffold showed good adhesive and self-healing properties. *In vivo* studies revealed that the scaffold enhanced skin regeneration and helped minimize scarring.

#### 5.5.3 Challenges associated with scaffold wound dressings

Scaffolds are a crucial factor in wound healing and tissue engineering (Yadav et al., 2023). However, their poor mechanical properties, potential cytotoxicity, and incongruous degradation rates limit their clinical application. Future research should focus on developing scaffolds according to each patient’s wound-specific factors. The use of functional nanoparticle hybrids and biocompatible natural polymers may improve mechanical properties and decrease degradation rates to satisfy the needs of various wound healing applications (Anitha et al., 2025).

## 6 Conclusion and future prospects

This review has highlighted recent advances in CS- and HA-based wound dressings for wound healing. CS has several excellent characteristics, including antibacterial, hemostatic, and antioxidant activity, which are crucial for facilitating wound healing. Similarly, HA, a component of the ECM, can provide a framework for nutrients or waste products, affect keratinocyte proliferation and migration, promote anti-inflammatory responses, increase angiogenesis, and provide antioxidant activity. At present, both CS and HA and their derivatives are used in commercial products for wound care. Wound dressings are based on hydrogels, nanofibers, films, sponges, and scaffolds. Hydrogel wound dressings maintain a moderately moist environment; absorb wound exudates; remove necrotic tissue; show good mechanical strength, biodegradability, and viscoelasticity; and provide excellent adhesion. Therefore, hydrogels based on polysaccharide are the most commonly used wound dressing material (Xue et al., 2025). Nanofiber wound dressings have a large specific surface area and a mimic the ECM structure, which is beneficial for hemostasis, surface functionalization, an optimal interaction between cells and nanofibers, microorganism inhibition, and nutrients permeation. Film wound dressings are flexible and, therefore, easily conform to the wound in different body areas—even the joints. The wound area covered by a film wound dressing can be inspected without removing the dressing, and films allow moderate moisture evaporation to alleviate pain. In addition, film dressings have demonstrated excellent bioactivity, such as swelling, tissue adhesion, and biodegradability. Sponge wound dressings have several positive characteristics, including high porosity, large surface area, elastic deformation, and 3D reaction environment. The tiny pore structure can be used to load drugs, as well as shield and shuttle them. Scaffold wound dressings can mimic the ECM, and thus, they provide an optimal niche for cells, shape and strengthen the wound bed, and enable the controlled release of bioactive factors for wound healing. Although there are distinct advantages associated with all these wound dressings, challenges persist in their clinical application, such as regulatory challenges, sterilization limitations, and high costs. For example, an HA-based hydrogel was prepared through physical crosslinking without chemical crossing agents by transforming an HA solution into an HA hydrogel through pH changes (Yang et al., 2026). The HA-based hydrogel showed desirable self-healing properties, excellent mechanical toughness, and good adhesiveness, with anti-inflammatory, antibacterial, immunoregulatory, and pro-angiogenic activity that expedited wound healing. However, the clinical translation of this pH-induced self-healing HA hydrogel faces several challenges. The hydrogel may prematurely degrade due to pH fluctuations in the wound area. Moreover, HA-based hydrogels have had significant differences among different production batches because of the high sensitivity of hydrogen bonds to temperature and ionic strength. Furthermore, the clinical regulatory process is undefined. Therefore, it is crucial to overcome these obstacles for successful clinical applications.

Biopolymer-based wound dressings are now widely used. In the near future, wound dressings will have superior biocompatibility, including excellent mechanical properties, degradation rates, and minimal to no toxicity. Moreover, they will incorporate drugs, wound healing proteins, and cells to meet the demand for biologically driven, regenerative medicinal approaches. Furthermore, smart wound dressings with physiological responsiveness will be developed to load drugs and control their release. In addition, personalized therapies will become more common with advances in proteomics, genomics, and stem cell technologies, potentially revolutionizing wound healing treatments (Ambekar and Kandasubramanian, 2019c; Christner et al., 1977; Han and Ceilley, 2017).
